# Determination of multiple drugs of abuse in human urine using dispersive liquid–liquid microextraction and capillary electrophoresis with PDA detection

**DOI:** 10.1080/20961790.2021.1986771

**Published:** 2021-12-09

**Authors:** Liang Meng, Shuhai Ye, Yilin Wu, Linda You

**Affiliations:** a Department of Forensic Science, Fujian Police College, Fuzhou, China; b Institute of Forensic Science, Fujian Provincial Public Security Department, Fuzhou, China

**Keywords:** Forensic sciences, forensic toxicology, dispersive liquid–liquid microextraction, capillary electrophoresis, drugs of abuse, urine sample

## Abstract

A new method was developed for pre-concentration and determination of multiple drugs of abuse in human urine using dispersive liquid–liquid microextraction (DLLME) and capillary electrophoresis (CE) with photodiode array detection. The method was based on the formation of tiny droplets of an organic extractant in the prepared sample solution using water-immiscible organic solvent (chloroform) dissolved in water-miscible organic dispersive solvent (isopropyl alcohol). The organic phase, which extracted eight drugs of abuse from the prepared urine solution, was separated by centrifugation. The sedimented phase was transferred into a small volume CE auto-sampler vial with 10 µL of 1% HCl methanol solution and evaporated to dryness. The residue was reconstituted in lidocaine hydrochloride (internal standard) aqueous solution and introduced by electrokinetic injection into CE. Under the optimum conditions, acceptable linear relationship was observed in the range of 3.0–500 ng/mL with the correlation coefficient (*r*) of 0.9982–0.9994 for spiked urine samples. The limit of detection (LOD) (S/N = 3) was estimated to be 1.0 ng/mL. A recovery of 75.7%–90.6% was obtained for spiked samples. The mean relative error (MRE) was within ±7.0% and the relative standard deviation (RSD) was less than 6.9%. The proposed DLLME-CE procedure offers an alternative analytical approach for the sensitive detection of drugs of abuse in real urine samples.

**Key points**
- The dispersive liquid-liquid microextraction (DLLME) was involved for the determination of drugs in urine with capillary electrophoresis with photodiode array detection (CE-PDA).
- Good linearity, sensitivity, recovery and precision were achieved.
- The proposed method was eco-friendly with microliter scale solvent consumption.

## Introduction

Fighting against drugs of abuse and addiction is an ongoing struggle for society and public health systems. To control these drugs (such as amphetamines and heroin) effectively, it is necessary to develop selective and sensitive analytical methods suitable for their unambiguous identification and determination in illicit samples and biological matrices. This has traditionally been carried out using gas chromatography-mass spectrometry (GC-MS) [1,2], high-performance liquid chromatography-mass spectrometry-mass spectrometry (HPLC-MSMS) [3,4], capillary electrophoresis (CE) [5,6], immunosensor [7] and immunoassay [8], etc. In recent years, the mixing use of drugs is becoming one of the epidemiological characteristics of drug abuse patterns [9]. The simultaneous screening and confirmation of multiple drugs in the body is of considerable importance for the investigation of intoxications, withdrawal and clinic treatment.

The structures of drugs and their metabolites are similar. They exist in complex matrices such as body fluids at different trace concentrations, so they are not easily determined both qualitatively and quantitatively. In this context, CE provides original characteristics in terms of separation mechanisms, rapid and efficient analysis with remarkably high resolution, small sample volume, high sample throughput, low operational cost and tolerance to biological matrices [10]. All these characteristics ideally make it an attractive technique in comparison with liquid chromatography (LC) and gas chromatography (GC) for forensic toxicological analysis [5,6]. However, poor concentration sensitivity of traditional CE with on-column UV detection due to low sample injection volume and short optical path length limit the use of CE as an effective method to determine trace analytes in biological and environmental samples [10].

To improve concentration detection limits in CE, several sample preparation techniques such as online sample concentration techniques [11,12], liquid–liquid extraction [13,14], and spectrometry (SPE) [15,16] have been developed, and these techniques have been applied to the forensic analysis [5,6,17–19]. Compared with the other sample preparation techniques, dispersive liquid–liquid microextraction (DLLME) has the advantages of low cost, excellent sample clean-up effect, and high extraction efficiency [20,21], which has been applied to the determination of trace analytes in various samples [22–25].

The aim of this study was to develop and validate a method to simultaneous screening and confirmation of multiple drugs of abuse in human urine using the combination of DLLME sample pretreatment and CE with photo diode array (PDA) analyte detection. Different parameters affecting the extraction process were studied and optimized in detail. The performance of the proposed method can provide higher stability and extraction efficiency in comparison with other extraction methods and was successfully employed to determine the trace level target analytes in human urine samples.

## Materials and methods

### Reagents and materials

6-Monoacetylmorphine (6-MAM) hydrochloride, morphine hydrochloride, codeine phosphate, methamphetamine hydrochloride, amphetamine hydrochloride, 3,4-methylenedioxymethamphetamine (MDMA) hydrochloride, 3,4-methylenedioxyamphetamine (MDA) hydrochloride and ketamine hydrochloride were purchased from Cerilliant (Merck KGaA, Darmstadt, Germany). The HPLC-grade methanol and acetonitrile were obtained from TEDIA Company (Fairfield, OH, USA). Ethanol, isopropyl alcohol, dichloromethane (CH_2_Cl_2_), chloroform (CHCl_3_), carbon tetrachloride (CCl_4_) and 1,1,2,2-tetrachloroethane (C_2_H_2_Cl_4_) were purchased from Kermel (Tianjin, China). Lidocaine hydrochloride (≥98%) was purchased from Sigma-Aldrich (St. Louis, MO, USA) for internal standard (IS). All other analytical grade reagents used for experiments were purchased from Kermel. All electrolytes and standard solutions were prepared with doubly distilled water. They were stored in the refrigerator at 4 °C, filtered through 0.45 µm disposable membrane filter (Millipore, Milford, MA, USA) and degassed by ultrasonication before use.

The blank urine samples were obtained from drug-free students in Fujian Police College. The real samples from the drug abusers were obtained from the Institution of Forensic Science (Fujian Provincial Public Security Department, Fuzhou, China) anonymously. Written informed consent was signed by all individuals for sample collection and used in this study. Ethical approval was obtained from by Fujian Police College Internal Review Board.

### Instrumentation

CE was performed with a CESI 8000 plus instrument equipped with a PDA detector (AB Sciex, Framingham, MA, USA). Separations were accomplished in a 75 µm I.D. fused silica capillary (Reafine, Handan, China) with an effective length of 50 cm (total length 60.3 cm). Detection was performed *via* on-capillary measurement of the UV absorptions at 214 nm. The measurements were performed at 25 °C.

### Sample preparation

To make the possible impurity precipitation, 0.5 mL of methanol and 10 mg zinc acetate were added into 1 mL of urine sample. Then a 3-min centrifugation at 10 625 ×*g* and 4 °C was carried out and the top aqueous layer was removed and filtered through a 0.45 µm membrane filter.

### DLLME

A 1.0 mL of prepared sample solution above was diluted with 1.0 mL of 30 mmol/L sodium tetraborate buffer (pH 9.2) and was placed in a 5 mL tube with conical bottom. The amount of 0.5 mL of isopropyl alcohol (disperser solvent) containing 41 µL of CHCl_3_ (extraction solvent) was injected rapidly into the sample solution by using a 1.00-mL syringe. A cloudy solution (water, isopropyl alcohol, and CHCl_3_) was formed in the test tube. In this step, the analytes were extracted into the fine droplets of CHCl_3_ in a few seconds. After centrifugation for 3 min at 10 625 ×*g*, the extraction solvent was sedimented in the bottom of the conical test tube (about (8.0 ± 0.2) µL). The sedimented phase (8 µL) was removed using a 10-µL GC microsyringe and placed into a small volume CE auto-sampler vial with 10 µL of 1% HCl methanol solution, then evaporated to dryness under nitrogen using an N-Evap evaporator (Organomation Assoc., Berlin, MA, USA) at room temperature. The residue was reconstituted in 20 µL of the 3 mg/L lidocaine hydrochloride aqueous solution and introduced by electrokinetic injection into CE.

### CE-UV analysis

Carrier electrolytes were prepared by neutralization of 100 mmol/L NaH_2_PO_4_ solution containing 20% methanol (v/v) with H_3_PO_4_ to the pH 4.0. Each new fused silica capillary was rinsed at 20 p.s.i. with 0.1 mol/L NaOH for 30 min, double distilled water for 30 min and buffer for 30 min. All samples were introduced by electrokinetic injection for 5 s at 5 kV. Each sample was injected only once. During analysis the instrument was operated at 25 kV, generating a current level of approximately 115 µA. Between electrophoretic separations the capillary was rinsed at 20 p.s.i. with double distilled water, 0.1 mol/L NaOH, double distilled water and the running buffer for 3 min, respectively.

### Method validation

Method validation was validated according to the published recommendations [26,27]. The linearity of the method was assessed in spiked samples. It was established from six calibration levels. For urine samples, the analytes at the concentration of 3.0, 10.0, 50.0, 100.0, 300.0 and 500.0 ng/mL were prepared, respectively. The spiked samples were treated in accordance with the above process (*n* = 5).

Based on the experimental data, the correlation of analytes concentration (*x*) with the peak area ratio of analytes to internal standard (*y*) was obtained. Linearity of the calibration curve was calculated using a line of best fit with correlation factor expected to be >0.99. Limit of detection (LOD) was calculated as 3 times of the signal to noise ratio (S/N). Lower limit of quantification (LLOQ) was calculated as 10 times of the S/N. The deviation of the measured concentration from the nominal concentration should be within ±15% except for the LLOQ level where *a* ± 20% deviation is acceptable.

Intra- and inter-day (over 5 days) precision and accuracy studies were investigated using quality control samples which were blank samples spiked with analytes at 10, 50 and 100 ng/mL (*n* = 5) for urine samples. Accuracy was expressed as a percentage mean relative error (MRE), which was deemed acceptable if the calculated concentrations fell within 20% of the concentration spiked. Precision was deemed acceptable if the percentage relative standard deviation (RSD) was <15%. Recovery percentage of the proposed DLLME procedure referred to comparing the peak area of the DLLME-treated analyte spiked sample with the pure analyte standard at the equivalent concentration without going through the DLLME process.

## Results and discussion

### Optimization of DLLME

Different parameters affecting the extraction efficiency such as the kind and volume of extraction and dispersive solvents, the pH of sample solution, and extraction time must be studied and optimized with the standard aqueous solution of the analytes. The enrichment factor (EF) and extraction recovery (ER) were used to assess the method optimized parameters as described by Rezaee et al. [28] and Leong et al. [29], as can be seen in the Supplementary Information.

CH_2_Cl_2_, CHCl_3_, CCl_4_ and C_2_H_2_Cl_4_ were pretested as extraction solvents to analyse the effect of the solvent on extraction efficiency. A series of sample solutions were studied by using 0.50 mL of methanol containing different volumes of extraction solvents to achieve 8.0 µL of settled phase [28], accordingly, 83.5, 50.0, 25.3, 20.0 µL of CH_2_Cl_2_, CHCl_3_, CCl_4_, and C_2_H_2_Cl_4_ were selected, respectively. As shown in Table 1, CHCl_3_ possessed the highest ER as compared with other extraction solvents.

**Table 1.** Enrichment recoveries obtained with the different extraction solvents evaluated for the extraction of drugs by dispersive liquid–liquid microextraction (DLLME).

Analytes	Enrichment recovery, mean±SD (*n* = 5)			
	Dichloromethane	Chloroform	Carbon tetrachloride	1,1,2,2-tetrachloroethane
Methamphetamine	46.0±2.3	67.0±1.1	10.0±2.9	50.6±1.8
Amphetamine	39.8±4.5	68.7±2.8	23.1±3.2	49.7±2.8
3,4-Methylenedioxymethamphetamine	49.6±3.3	70.6±3.4	30.4±3.3	67.4±3.2
3,4-Methylenedioxyamphetamine	50.2±2.6	72.0±1.8	16.4±3.0	53.3±1.8
Ketamine	74.0±3.0	80.0±2.6	26.0±3.4	73.0±2.8
Codeine	81.4±1.9	87.0±1.7	31.7±2.8	80.9±2.1
Morphine	82.4±2.1	88.9±2.8	36.0±3.3	81.6±2.8
6-Monoacetylmorphine	81.2±2.9	87.1±3.0	29.9±3.4	80.1±2.8

Extraction conditions: water sample volume, 5.0 mL; pH of sample, 9.0; dispersive solvent, 0.50 mL methanol; extraction solvents, 83.5 µL CH_2_Cl_2_, 50.0 µL CHCl_3_, 25.3 µL CCl_4_, 20.0 µL C_2_H_2_Cl_4_; room temperature; analytes concentration spiked, 10 ng/mL; internal standard, 4 µg/mL lidocaine.

**Figure 1.**
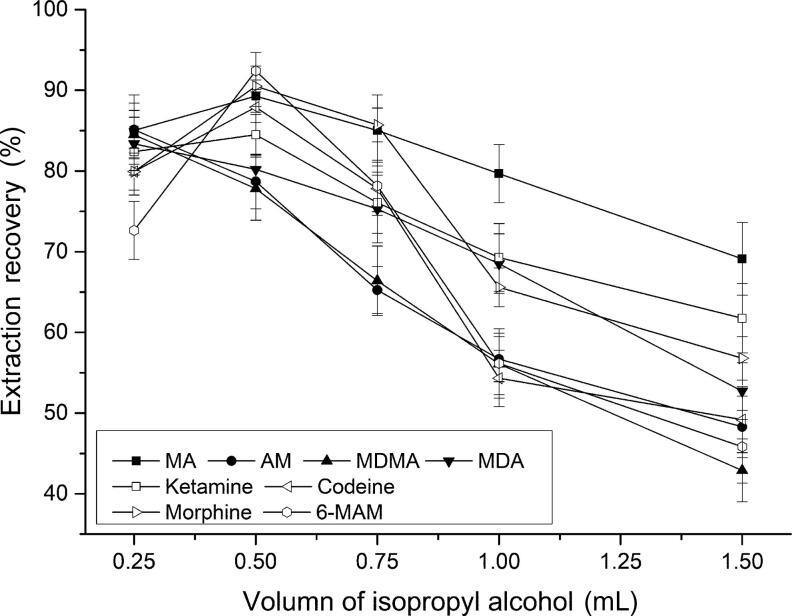
Effect of the dispersive solvent (isopropyl alcohol) volume on extraction recovery. Extraction conditions: aqueous sample volume, 1.0 mL; pH of sample, 9.0; dispersive solvent, isopropyl alcohol; extraction solvent, 41.0 μL CHCl_3_; room temperature; analytes concentration spiked, 50 ng/mL; internal standard, 3 µg/mL lidocaine. MA: methamphetamine; AM: amphetamine; MDMA: methylenedioxymethamphetamine; MDA: methylenedioxyamphetamine; MAM: monoacetylmorphine.

Methanol, acetonitrile, ethanol and isopropyl alcohol were tested as disperser solvent. To confirm the constant volume of the sedimented phase was constant (8.0 µL), the experiments were performed using 0.5 mL of methanol, acetonitrile, ethanol and isopropyl alcohol containing 50.0, 48.0, 20.8 and 41.0 µL of CHCl_3_, respectively. The results indicated that isopropyl alcohol exhibited the highest extraction efficiency (Table 2). Subsequently, various experiments were performed to optimize the volume of dispersive solvent by using different volumes of isopropyl alcohol (0.25, 0.50, 0.75, 1.00 and 1.50 mL) containing 17.0, 41.0, 54.4, 65.8 and 81.6 µL CHCl_3_, respectively. As shown in Figure 1, 0.5 mL of isopropyl alcohol containing 41.0 µL CHCl_3_ gave a good extraction performance.

**Table 2.** Enrichment recoveries obtained with the different dispersive solvents evaluated for the extraction of drugs by dispersive liquid–liquid microextraction (DLLME).

Analytes	Enrichment recovery, mean±SD (*n* = 5)			
	Methanol	Acetonitrile	Ethanol	Isopropyl alcohol
Methamphetamine	67.0±1.1	56.8±1.0	70.7±2.5	89.2±2.0
Amphetamine	68.7±2.8	59.3±1.9	23.1±3.2	78.7±3.4
3,4-Methylenedioxymethamphetamine	70.6±3.4	62.6±3.3	30.4±3.3	77.8±3.9
3,4-Methylenedioxyamphetamine	72.0±1.8	66.7±2.8	71.8±3.0	80.2±1.7
Ketamine	80.0±2.6	80.9±2.6	76.9±3.9	84.5±2.5
Codeine	87.0±1.7	83.8±2.7	85.6±2.2	87.9±1.9
Morphine	88.9±2.8	80.4±2.9	87.8±3.1	90.5±2.5
6-Monoacetylmorphine	87.1±3.0	82.1±3.5	87.9±3.2	92.4±2.3

Extraction conditions: water sample volume, 5.0 mL; pH of sample, 9.0; dispersive solvent, 0.50 mL methanol, acetonitrile, ethanol, isopropyl alcohol; extraction solvents, 50.0, 48.0, 20.8, 41.0 µL CHCl_3_; room temperature; analytes concentration spiked, 10 ng/mL; internal standard, 4 µg/mL lidocaine.

**Figure 2.**
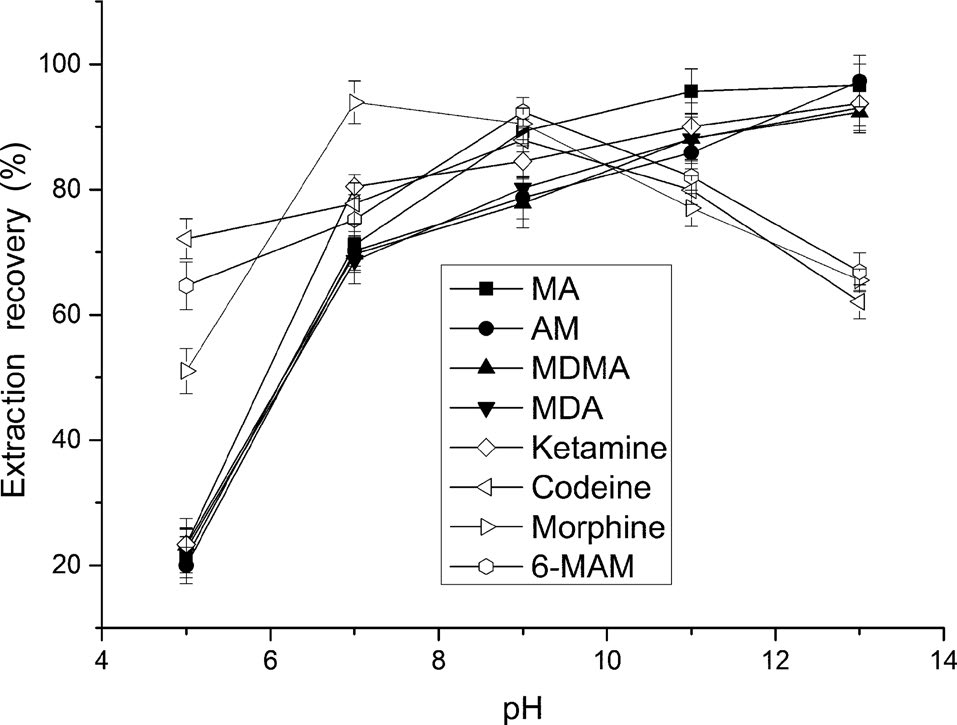
Effect of the pH of sample solution on extraction recovery. Extraction conditions: aqueous sample volume, 1.0 mL; dispersive solvent, 0.5 mL isopropyl alcohol; extraction solvent, 41.0 µL CHCl_3_; room temperature; analytes concentration spiked, 50 ng/mL; internal standard, 3 µg/mL lidocaine. MA: methamphetamine; AM: amphetamine; MDMA: methylenedioxymethamphetamine; MDA: methylenedioxyamphetamine; MAM: monoacetylmorphine.

The effect of sample pH was tested in the pH range from 5 and 13. As shown in Figure 2, the ERs of amphetamines and ketamine were maximized at pH 11 and then slightly increased. However, a gradual increase of the ERs of heroin metabolites was observed with increasing sample pH, up to 9.0, then decreased, which was attribute to the amphoteric characteristic of morphine. Then, the pH 9.0 was selected for the sample solution. The desired pH was achieved by diluting with sodium tetraborate buffer (pH 9.2) to the prepared sample solution.

Previous studies [26–29] indicated that the extraction quickly reached equilibrium and the DLLME method is time-independent. However, the good reproducibility cannot be ensured in an extraction time as short as a few seconds. Consequently, 1 min of extraction time was chosen in the following experiments.

### Method validation

In order to proceed with the current evaluation of the proposed DLLME technique, linearity, LOD, and repeatability were investigated under optimized conditions with the spiked samples. The performance of the developed procedure is summarized in Table 3. The calibration curve was linear for concentrations of all analytes in the range of 3.0–500 ng/mL with the correlation coefficient ranging from 0.9982 to 0.9994 for spiked urine samples. The LOD (S/N = 3) was estimated to be 1.0 ng/mL. The LLOQ (S/N = 10) was estimated to be 3.0 ng/mL. The recoveries vary from 75.7% to 90.6% with RSD ≤6.9%. Precision and accuracy at the three concentration levels were determined to be satisfactory. The repeatability was acceptable and comparable with other methods reported in the literature (Table 4). The results suggested that the methods had high sensitivity and reproducibility, which could be applied to monitoring multiple drugs of abuse in real samples by DLLME-CE.

**Table 3.** The performance of the proposed methods in spiked urine samples.

Analytes	Linear dynamic range (ng/mL)	*r*	LOD (ng/mL)	Added (ng/mL)	Recovery (%)	RSD (%), *n* = 5		MRE (%), *n* = 5	
						Intra-day^a^	Inter-day^b^	Intra-day^a^	Inter-day^b^
MA	6.0–500	0.9982	2.0	10	88.2	6.7	4.9	6.1	5.8
				50	86.1	5.7	4.9	5.5	5.6
				100	85.7	5.3	5.2	5.8	5.2
AM	6.0–500	0.9986	2.0	10	75.7	4.5	6.2	5.4	6.6
				50	79.1	6.4	6.7	4.9	5.9
				100	88.5	6.0	5.2	5.3	4.8
MDMA	6.0–500	0.9984	2.0	10	87.9	4.8	4.7	6.0	4.9
				50	86.5	4.9	5.9	5.3	6.5
				10	86.1	5.7	5.3	5.6	3.9
MDA	6.0–500	0.9984	2.0	10	78.5	5.6	5.4	4.4	5.0
				50	80.2	6.0	5.6	4.7	5.9
				100	87.7	5.0	6.1	5.8	3.9
Ketamine	3.0–500	0.9990	1.0	10	85.0	6.5	4.9	5.4	4.8
				50	89.0	5.9	3.8	4.1	4.6
				100	90.6	6.1	5.0	6.6	5.5
Codeine	3.0–500	0.9994	1.0	10	81.0	5.8	4.3	4.9	6.1
				50	84.0	4.8	4.1	3.8	4.7
				100	83.4	3.4	3.8	6.4	6.5
Morphine	3.0–500	0.9990	1.0	10	85.8	6.1	3.5	5.7	5.5
				50	87.7	5.3	5.7	4.5	5.0
				100	89.4	6.9	3.4	7.0	4.5
6-MAM	3.0–500	0.9991	1.0	10	89.0	5.3	5.1	6.4	4.9
				50	85.8	5.1	3.8	6.2	5.8
				100	90.1	4.3	5.5	5.3	5.1

^a^Intra-day repeatability was calculated by analysing spiked samples within 1 day.

^b^Inter-day repeatability was calculated by analysing spiked samples over a period of 5 days.

LOD: limit of detection; RSD: relative standard deviation; MRE: mean relative error; MA: methamphetamine; AM: amphetamine; MDMA: methylenedioxymethamphetamine; MDA: methylenedioxyamphetamine; MAM: monoacetylmorphine.

**Table 4.** Comparison of DLLME with SPME and LPME for determination of MA and morphine in aqueous solution.

Analytes	Method	Linearity (ng/mL)	LOD (ng/mL)	RSD (%, *n* = 5)
MA	DLLME-CE-UV (This method)	6.0–500	2.0	<6.7
	SPME-HPLC-fluorescence [30]	1 000–10 000	100	<14
	LPME-FIA-APCI-MSMS [31]	–	30	–
Morphine	DLLME-CE-UV (This method)	3.0–500	1.0	<7.0
	LPME-HPLC-UV [32]	50–500	20	<8.2

DLLME: dispersive liquid–liquid microextraction; SPME: solid-phase microextraction; LPME: liquid-phase microextraction; MA: methamphetamine; LOD: limit of detection; RSD: relative standard deviation; CE: capillary electrophoresis; UV: ultra violet; HPLC: high performance liquid chromatography; FIA: flow-injection analysis; APCI: atmospheric pressure chemical ionization; MSMS: mass spectrometry-mass spectrometry.

### Analysis of real biological samples

**Figure 3.**
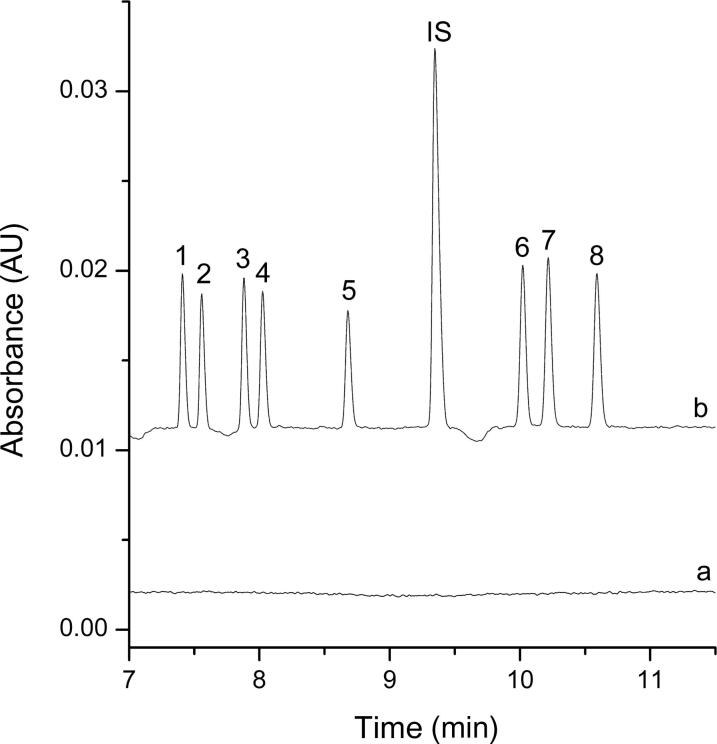
Electropherograms obtained for the spiked biological samples before (a) and after (b) dispersive liquid–liquid microextraction (DLLME) extraction under the optimum conditions. Extraction conditions: dispersive solvent, 0.5 mL isopropyl alcohol; extraction solvent, 41.0 µL CHCl_3_; room temperature; pH of sample, 9.0; analytes concentration spiked, urine: 50 ng/mL; internal standard: 3 µg/mL lidocaine. Peak identification: (IS) lidocaine, (1) AM: amphetamine, (2) MA: methamphetamine, (3) MDA: methy­lenedioxyamphetamine, (4) MDMA: methylenedioxymethamphetamine, (5) ketamine, (6) codeine, (7) morphine, (8) 6-MAM: 6-monoacetylmorphine.

**Figure 4.**
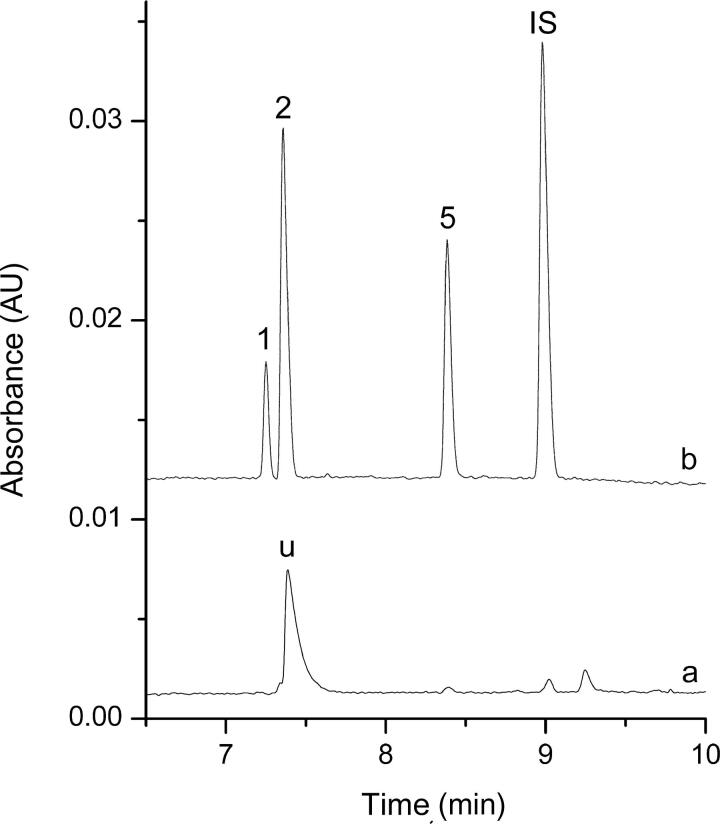
Electropherograms obtained for the real biological samples from the drug abuser before (a) and after (b) dispersive liquid–liquid microextraction (DLLME) extraction under the optimum conditions. Extraction conditions: dispersive solvent, 0.5 mL isopropyl alcohol; extraction solvent, 41.0 μL CHCl_3_; room temperature; pH of sample, 9.0; analytes concentration spiked, urine: 50 ng/mL; internal standard: 3 μg/mL lidocaine. Peak identification: (IS) lidocaine, (1) AM: amphetamine, 42.1 ng/mL, (2) MA: methamphetamine, 102.6 ng/mL, (5) ketamine, 84.7 ng/mL.

The proposed DLLME-CE procedure described above was applied to determine the multiple drugs of abuse in spiked and real samples. In addition to the high preconcentration, electropherograms obtained for the spiked biological samples and the real biological samples after DLLME extraction demonstrated a substantial sample clean-up effect. Electropherograms of these samples before and after DLLME extraction were shown in Figures 3 and 4, no other peaks were present in the electropherograms, and high preconcentration was obtained after DLLME extraction.

## Conclusion

This study has demonstrated the successful application of DLLME-CE-UV method in the determination of multiple drugs of abuse in human urine. The optimum conditions of extraction performance have been obtained. The experimental results reveal that this method provides high extraction efficiency within a short time, good selectivity, low LODs and good linearity over the investigated concentration range. The performance of this procedure in the extraction of 6-MAM, morphine, codeine, MA, AM, MDMA, MDA and ketamine from human urine was satisfactory. Compared with SPME and LPME, besides the short extraction time, low cost, and feasibility, the best advantages of the presented method are the high sensitivity and good linearity. Therefore, it has the potential to be a powerful tool for the analysis of drugs of abuse in forensic investigations.

## Supplementary Material

Supplemental Material
