# Supplementary material for: Determination of multiple drugs of abuse in human urine using dispersive liquid–liquid microextraction and capillary electrophoresis with PDA detection
Source: Forensic Sci Res. 2021 Dec 9;7(2):265–71. doi: 10.1080/20961790.2021.1986771 (PMC9245984; doi:10.1080/20961790.2021.1986771)
Supplement: Supplemental Material [file TFSR_A_1986771_SM7894.docx]

**Supplementary Information for**

**Determination of multiple drugs of abuse in human urine and hair using dispersive liquid-liquid microextraction and capillary electrophoresis with PDA detection**

Liang Meng,

**1. Calculation of enrichment factor and extraction recovery**

The enrichment factor (EF) and extraction recovery (ER) were used to assess the method optimized parameters.

The EF was defined as the ratio between the analyte concentration in the sedimented phase (*C_sed_*) and the initial concentration of analyte (*C_0_*) within the sample:

$$EF=\frac{C_{sed}}{C_{0}}$$

The sedimented phase was reconstituted in 20 μl of 3 mg/L lidocaine hydrochloride aqueous solution. The concentration of reconstituted analyte was obtained from calibration graph of direct injection of drugs standard solution at the range of 3.0 - 500 ng/mL.

The ER was defined as the percentage of the total analyte amount (*n_0_*) which was extracted to the sedimented phase (*n_sed_*).

$$ER=\frac{n_{sed}}{n_{0}}\times100=\frac{C_{sed}\times V_{sed}}{C_{0}\times V_{aq}}\times100=\left(\frac{V_{sed}}{V_{aq}}\right)EF\times100$$

where *V_sed_* and *V_aq_* are the volumes of sedimented phase and sample solution, respectively.
